# Skeletal muscle atrophy induced by aging and disuse atrophy are strongly associated with the upregulation of the endoplasmic stress protein CHOP in rats

**DOI:** 10.1007/s11033-025-10415-4

**Published:** 2025-03-18

**Authors:** J. Max Michel, Joshua S. Godwin, Nathan R. Kerr, Thomas E. Childs, Frank W. Booth, C. Brooks Mobley, David C. Hughes, Michael D. Roberts

**Affiliations:** 1 School of Kinesiology, Auburn University, Auburn, AL USA; 2 Department of Biomedical Sciences, University of Missouri, Columbia, MO USA; 3 Department of Medical Pharmacology and Physiology, University of Missouri, Columbia, MO USA; 4 Dalton Cardiovascular Research Center, University of Missouri, Columbia, MO USA; 5 Aging and Metabolism Research Program, Oklahoma Medical Research Foundation, Oklahoma City, OK USA; 6 Edward Via College of Osteopathic Medicine, Auburn, AL USA; 7 School of Kinesiology Director, Nutrabolt Applied and Molecular Physiology Laboratory, Auburn University, 301 Wire Road, Office 286, Auburn, AL 36849 USA

**Keywords:** Endoplasmic reticulum stress, Unfolded protein response, CHOP, Aging, Disuse

## Abstract

**Background:**

While canonical anabolic and proteolytic pathways have been well examined in the context of skeletal muscle proteostasis, the roles of endoplasmic reticulum stress (ERS) and the induced unfolded protein response (UPR) are underappreciated. Thus, we aimed to determine whether aging and/or disuse atrophy in rats altered skeletal muscle ERS/UPR markers.

**Methods and Results:**

Soleus (SOL) and plantaris (PLT) muscles of 3-month-old (mo), 6 mo, 12 mo, 18 mo, and 24 mo rats (9–10 per group, 48 in total) were analyzed for UPR proteins with further analysis performed on the protein CHOP. The gastrocnemius muscles of 4 mo rats that had undergone hindlimb immobilization (HLI, *n* = 12) or sham casting (CTL, *n* = 12) were analyzed for similar targets as well as more extensive CHOP-related targets. CHOP protein was greater in the PLT and SOL of 18 and 24 mo rats versus other age groups (*P* < 0.05). Moreover, negative correlations existed between CHOP expression and normalized PLT (*R*=-0.702, *P* < 0.001) and SOL (*R*=-0.658, *P* < 0.001) muscle weights in all rats analyzed at different ages. CHOP protein expression was also greater in the gastrocnemius of HLI versus CTL rats (*P* < 0.001), and a negative correlation existed between CHOP protein expression and normalized muscle weights in these rats (*R*=-0.814, *P* < 0.001). Nuclear CHOP protein levels (*P* < 0.010) and genes transcriptionally regulated by CHOP were also greater in HLI versus CTL rats (*P* < 0.001) implicating transcriptional activity of CHOP is elevated during disuse atrophy.

**Conclusions:**

CHOP is operative during aging- and disuse-induced skeletal muscle atrophy in rodents, and more research is needed to determine if CHOP is a key mechanistic driver of these processes.

## Introduction

Skeletal muscle is the largest tissue by weight in the human body and exhibits remarkable plasticity [1, 2]. This hallmark of skeletal muscle is exemplified by its ability to respond to different stimuli [3]. Common adaptations include hypertrophy in response to mechanical overload and atrophy in response to whole body disuse or limb unloading [4–6]. Aging along with disuse are other potent drivers of skeletal muscle (mal)adaptation [2, 7], often manifesting in dysregulated proteostasis, increased senescent burden, and increased endoplasmic reticulum (ER) stress (ERS) [8, 9]. While alterations to muscle protein synthesis (MPS) and muscle protein breakdown (MPB) are well studied in the context of aging, the impact of ERS is an underappreciated mechanism of skeletal muscle regulation [10].

The ER is a primary site of protein folding and the release of proteins from the ER is a highly stringent process. Indeed, release of proteins from the ER is thought to be the rate limiting step in the arrival of mature proteins at their subsequent destinations (e.g. at the plasma membrane or in circulation; reviewed in [11]). This phenomenon is indicative of the proper structure conformity that proteins must acquire within the ER prior to release and performance of canonical actions. ERS refers to the stress imposed upon the ER in response to an increase in truncated, improperly translated, or otherwise misfolded proteins, and the resultant pathway that becomes active in response to sustained ERS is the unfolded protein response (UPR) [12, 13]. The UPR is a signaling cascade triggered by three main effectors including protein kinase R (PKR) like ER kinase (PERK), inositol requiring enzyme 1α (IRE1α), and activating transcription factor 6 (ATF6). The UPR serves to blunt global protein synthesis via phosphorylation of eukaryotic initiation factor 2α (eIF2α) and preferentially translate a set of basic leucine zipper motif (bZIP) transcription factors (e.g. Activating Transcription Factor 4 (ATF4), spliced form of X-box binding protein 1 (XBP1s)). These bZIP transcription factors enhance the translation of chaperones, folding enzymes, and ER-associated protein degradation (ERAD) enzymes that are beneficial in mitigating the burden of errant proteins [14].

The UPR can be beneficial for skeletal muscle adaptation by effectively serving as a brake on translation during periods of high concentrations of misfolded proteins, thus allowing the cell to regain translational control and produce properly functioning proteins. This response can, however, become maladaptive if the unfolded protein burden is not remediated, as sustained activation of this system can trigger apoptosis and cell death via the upregulation of proapoptotic proteins such as the terminal effector C/EBP homologous protein (CHOP) [12, 14]. Indeed, the downstream induction of CHOP has been shown to play a major role in the proapoptotic function of the UPR [15–17]. ERS is often observed in the context of disease and/or intentional provocation (e.g. cancer models, tunicamycin treatment, high glucose or type 2 diabetes mellitus models) and can interplay with other cell outcomes such as increases in senescence or increased proteolysis [18–23]. Given that ERS can enhance the burden imposed by other markers typically associated with dysfunction in aging [24] it has become clear that ERS can be harmful to longevity [25–27].

While ERS burden and subsequent activation of the UPR is often elevated in aged (22 + months of age) versus young (3–6 months of age) rodents, UPR markers at different ages do not appear to have been examined [12]. Therefore, the purpose of this study was to examine the UPR response in skeletal muscle in the basal state from a cohort of Fischer 344 rats that were collected from young to old age. As a secondary aim, we sought to examine similar markers in response to hindlimb immobilization (HLI) induced skeletal muscle atrophy in a cohort of adult Wistar rats to determine if HLI elicited similar effects.

## Materials and methods

### Animal experimental procedures

All procedures involving animal husbandry and experimentation for rats collected at different ages were approved by Auburn University’s Institutional Animal Care and Use Committee (protocol #2015–2790). Male, Fischer 344 rats (300–600 g) aged 3, 6, 12, 18, and 24 months (*n* = 9–10 rats per group) were purchased from Envigo Laboratories (Indianapolis, IN, USA), and housed two per cage on the week prior to and during experimentation. During this time, animal quarters were maintained on a constant 12 h light: 12 h dark cycle at ambient room temperature and tap water and standard rodent chow (24% protein, 58% carbohydrate, 18% fat; Teklad Global #2018 Diet, Envigo Laboratories) were provided to animals *ad libitum.* The morning of experimentation, animals were removed from their quarters between 0600 and 0700, transported to the Molecular and Applied Sciences Laboratory in the School of Kinesiology building and euthanized under CO2 gas induction in a 2 L chamber (VetEquip, Inc., Pleasanton, CA, USA). Following euthanasia, body masses were recorded, right-leg plantaris and soleus muscles were dissected out, and muscles were weighed using an analytical scale with a sensitivity of 0.0001 g (Mettler-Toledo; Columbus, OH, USA). During dissection muscles were cut in very close proximity at the origin and insertion sites and visible connective tissue at the insertion site was removed. Muscles were then processed for biochemical assays on the day of euthanasia as described in the following paragraphs.

All procedures involving animal husbandry and experimentation for rats undergoing hindlimb immobilization were approved by the University of Missouri Animal Care and Use Committee (MU ACUC) (ACUC protocol #35961). Three-month-old female Wistar rats (*n* = 12 per group) were ordered from Charles River Laboratories for this study. Prior to the start of the study, all rats were housed until ~ 15 weeks of age when groups were chosen at random. For rats undergoing hindlimb immobilization (HLI), veterinary casts were applied (BSN Medical Delta-Lite Plus Casting Tape). Briefly, rats were anesthetized using isoflurane and casting material was applied to the extended hindlimbs with feet positioned in plantarflexion and knee at or near full extension. Rats in the CTL group were either completely cast-free or had the same casting material wrapped around the abdomen to mimic casting variables without physiological disuse. Notably, these CTL groups were collapsed due to no differences in downstream experiments between the two control groups. Rats were housed within-group in large cages to maintain socialization and mobility. More details regarding general animal husbandry can be found in Kerr et al. [28]

Details regarding euthanasia procedures can be found in Kerr et al. [28] Briefly, upon the tenth day of hindlimb disuse, a ketamine/xylazine cocktail was administered to facilitate cast removal and sacrifice. Then the 4-month-old rats were euthanized via decapitation whereafter the gastrocnemius was dissected out and weighed on an analytical scale, then immediately flash-frozen in liquid nitrogen. Gastrocnemius muscles were then stored at -80 °C for subsequent analysis.

Critically, we were limited to the use of male Fischer 344 rats for aging experiments and female Wistar rats for disuse-induced atrophy experiments. We acknowledge that there is likely an effect of sex and strain of these rats of the results reported herein, however it has been reported that both male and female rats experience significant atrophy in response to aging and hindlimb disuse regardless of strain.

### Wet laboratory analyses

#### Real-time qPCR

Frozen muscle foils were removed from − 80ºC storage and crushed on a liquid nitrogen-cooled ceramic mortar and pestle. Approximately 10 mg of muscle was used to isolate RNA via the Trizol (Thermo Scientific, Waltham, MA, USA) method coupled with the Direct-zol RNA miniprep kit (Zymo Research, Irvine, CA, USA) per the manufacturer’s recommendations. Following RNA isolation, the RNA pellet was reconstituted in 20 µL of RNase-free water and RNA concentrations were determined in duplicate at an absorbance of 260 nm by using a NanoDrop Lite (Thermo Scientific, Waltham, MA, USA). Thereafter, cDNA (2 µg) was synthesized using a commercial qScript cDNA SuperMix (Quanta Biosciences, Gaithersburg, MD, USA) per the manufacturer’s recommendations.

qPCR was performed with gene-specific primers and SYBR-green-based methods (Quanta Biosciences) using a real-time PCR thermal cycler (Bio-Rad). Gene-specific primers were designed with primer design software (Primer3Plus, Cambridge, MA, USA). The final volume of qPCR reactions was 20 µL, which contained a final concentration of 2 µM of forward and reverse primers and 25 ng of cDNA. All reactions were performed in duplicate. Forward and reverse sequences for all primers are shown in Table 1. Fold-change values from CTL were performed using the 2^ΔΔCq^ method where 2^ΔCq^ = 2^ [housekeeping gene (HKG) Cq– gene of interest Cq], and 2^ΔΔCq^ (or fold-change) = [2^ΔCq^ individual CTL or HLI value/2^ΔCq^ average of all PRE values]. Primer sets for valosin-containing protein (VCP) were used for mRNA normalization given that this gene did not differ between rat groups (*P* > 0.05).

**Table 1:** Rat primer sequences used for qPCR

Primer	Forward Primer Sequence (5’ ◊ 3’)	Reverse Primer Sequence (5’ ◊ 3’)
PPP1R15A (GADD34)	ATTTCCTTGCTGTCGGGCA	TCGATCTCGTGCAAACTGCT
BAX	GAACCATCATGGGCTGGACA	CCGAAGTAGGAAAGGAGGCC
VCP (HKG)	AGACCCAACAGCATTGACCC	CGTCCTGTAGCATCAGGGAT

Abbreviations: PPP1R15A, Protein phosphatase 1 regulatory subunit 15 A; BAX, BCL-2 associated X, apoptosis regulator; VCP, Valosin-containing protein; HKG, Housekeeping gene for data normalization

### Western blotting

During tissue extraction for RNA isolation a portion of skeletal muscle tissue was also allocated for protein isolation. Approximately 20 mg of tissue was placed in 1.7 mL microcentrifuge tubes and homogenized in general cell lysate buffer (VWR; Cat #: 97063-130) using tight-fitting pestles. Tissues were then centrifuged at 500 x g for 10 min, whereafter supernatants were collected for further analysis.

Protein lysates were prepared via homogenization with tight fitting pestles in 500 µL of general cell lysis buffer (catalog #: 9803 S; Cell Signaling Technologies) followed by centrifugation at 500 G at 4 °C. Lysates were then batch process-assayed for total protein content using a BCA Protein Assay Kit (Thermo Fisher; Waltham, MA, USA). Lysates were then prepared for western blotting using 4x Laemmli buffer at 1 µg/µL. Thereafter, 15 µL of prepped samples were loaded onto 4–15% SDS-polyacrylamide gels (Bio-Rad, Hercules, CA, USA) and subjected to electrophoresis (180 V for 50 min) using pre-made 1x SDS-PAGE running buffer (VWR; Cat #: 0783). Proteins were then transferred (200 mA for 2 h) to polyvinylidene difluoride membranes (Bio-Rad), Ponceau stained and imaged to ensure equal protein loading between lanes. Membranes were then blocked for 1 h at room temperature with 5% nonfat milk powder in Tris-buffered saline with 0.1% Tween-20 (TBST; VWR). Membranes containing 1-hour treated samples were incubated with the antibodies described in Table 2 at a 1:1000 dilution in TBST with 5% bovine serum albumin (BSA) overnight. The following day, membranes were incubated with horseradish peroxidase-conjugated HRP-conjugated anti-rabbit IgG (Cell Signaling Technology, Cat No. 7074), HRP-conjugated anti-mouse IgG (Cell Signaling Technology, Cat No. 7076), or HRP-conjugated anti-goat IgG (Genetex, Irvine, CA, USA Cat No. GTX628547-01) in TBST with 5% BSA at room temperature for 1 h. Membrane development was performed using an enhanced chemiluminescent reagent (Luminata Forte HRP substrate; Millipore Sigma), and band densitometry was performed using a gel documentation system and associated densitometry software (ChemiDoc Touch, Bio-Rad). Densitometry values of protein targets were normalized to Ponceau densities. These values were then normalized to the grand mean of the appropriate control to obtain relative protein expression. These values were then expressed as fold change from control, where control was set to 1.00. Phosphorylated target band densities were divided by pan densities of the same target to obtain a ratio.

**Table 2:** *Antibodies used for Western blotting*

Antibody (anti-target)	Host Species	Catalog Number	Manufacturer
BiP	Rabbit	3183	Cell Signaling Technology
Xbp1s	Rabbit	40,435	Cell Signaling Technology
ATF6	Mouse	ALX-804-381	Enzo Life Sciences
ATF4	Goat	ab1371	Abcam
Phospho-eIF2α (Ser51)	Rabbit	3398	Cell Signaling Technology
eIF2α	Rabbit	5324	Cell Signaling Technology
CHOP	Mouse	2895	Cell Signaling Technology
Phospho-JNK (Thr183/Tyr185)	Mouse	9255	Cell Signaling Technology
JNK	Rabbit	9252	Cell Signaling Technology

Abbreviations: BiP, Binding immunoglobulin protein; Phospho, phosphorylated; Xbp1s, X-box binding protein 1; ATF6, Activating transcription factor 6; ATF4 Activating transcription factor 4; eIF2α, Eukaryotic initiation factor 2 alpha; CHOP, CCAAT/enhancer-binding protein (C/EBP) homologous protein; JNK, c-Jun N-terminal kinase

### Nuclear protein fraction isolation

Nuclear and sarcoplasmic fractions were separated from HLI and CTL rats via a commercially available kit (ab113474; Abcam; Cambridge, UK) by adhering to manufacturer’s instructions. After isolations, BCA assays were performed to determine protein concentrations in each fraction and western blotting was performed as described above.

### Statistics

Statistical analyses were performed using GraphPad Prism (Version 10.1.1; GraphPad Software, San Diego, CA, USA). All data are presented as means and standard deviation (SD), and individual respondent data are also presented.

Rat aging data were checked for normality using the Shapiro-Wilk test. For normally distributed data, one-way repeated measures ANOVAs were performed. In cases where the ANOVA showed significance (*P* < 0.05), Tukey *post hoc* tests were performed between age groups. For non-normally distributed data, Friedman’s tests were performed. In cases where the Friedman’s test demonstrated significance, a Wilcoxon’s signed rank test was performed between age groups.

Hindlimb disuse rat data were checked for normality using the Shapiro-Wilk test. For normally distributed data, unpaired t-tests were performed with significance being established as *P* < 0.05. For non-normally distributed data, Mann-Whitney tests were performed with significance level again set at *P* < 0.05.

Given the substantial findings suggesting that CHOP plays a role in the proapoptotic response, we opted to further investigate this protein in the context of aging- and disuse-atrophy. Therefore, correlations were performed to examine the relationship between CHOP expression and muscle size changes in all rats examined herein. Data for both aging and hindlimb immobilization rats were checked for normality and since all failed to meet normality assumptions, Spearman’s correlations were performed between CHOP protein expression and normalized muscle weight ((muscle of interest/Body weight)*100). Associations were considered significant if *P* < 0.05.

## Results

### Rodent characteristics

For male Fischer 344 rats analyzed in aging experiments, right PLT weight (in mg) normalized to body weight (in g) was 0.86 ± 0.03 mg/g for 3 mo rats, 0.90 ± 0.03 mg/g for 6 mo rats, 0.79 ± 0.04 mg/g for 12 mo rats, 0.73 ± 0.04 mg/g for 18 mo rats, and 0.70 ± 0.05 mg/g for 24 mo rats. Significant differences (*P* < 0.05) in values were noted whereby 3 mo and 6 mo were greater than other age groups and 12 mo were greater than 18 mo and 24 mo. In these same rats, the right SOL weight (in mg) normalized to body weight (in g) showed a similar pattern, where values were 0.36 ± 0.03 mg/g for 3 mo, 0.32 ± 0.05 mg/g for 6 mo, 0.34 ± 0.02 mg/g for 12 mo, 0.28 ± 0.03 mg/g for 18 mo, and 0.30 ± 0.04 for 24 mo rats. Again, significant differences (*P* < 0.05) in values were noted whereby 3 mo were greater than 6 mo, and 18 and 24 mo values were lower than other age groups. For female Wistar rats in the disuse experiment, gastrocnemius weight (in mg) normalized to body weight (in g) was 3.52 ± 0.33 mg/g for HLI rats and 5.30 ± 0.27 mg/g for CTL rats. This 33.6% difference reached statistical significance (*P* < 0.001). Readers are referred to Mobley et al. [29] and Kerr et al. [28] for more detailed phenotypic data for rat aging and hindlimb immobilization analyses, respectively.

### ERS markers with aging in the plantaris muscle

**Fig. 1:**
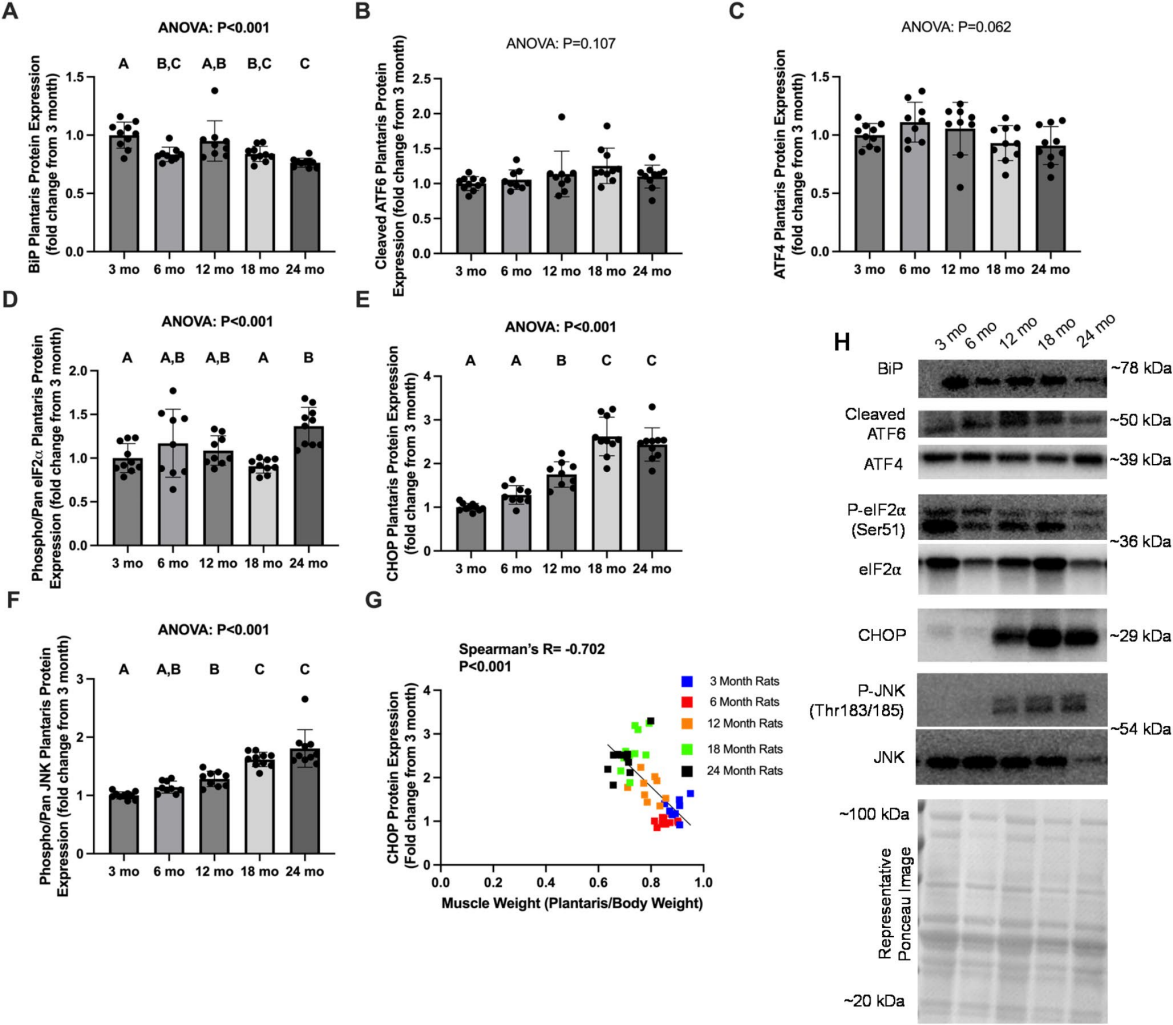
Endoplasmic Reticulum Stress related protein expression in the plantaris of Fischer 344 with aging. Legend: Data are presented as individual data points with standard deviation as error bars (panels **A**-**F**), or individual data points with representative line of best fit (panel **G**). Results are from the plantaris muscles of Fischer 344 rats sacrificed at 3 mo (*n* = 10), 6 mo (*n* = 9), 12 mo (*n* = 9), 18 mo (*n* = 10), and 24 mo (*n* = 10) for protein expression of BiP (panel **A**), cleaved ATF6 (panel **B**), ATF4 (panel **C**), phosphorylated/pan eIF2α (panel **D**), CHOP (panel **E**), phosphorylated/pan JNK (panel **F**), and the association between CHOP and normalized muscle weight [(plantaris/body weight)*100] (panel **G**). Representative western blot and Ponceau stain images are included (panel **H**). Western blot band densities are normalized to ponceau densities. ANOVA P values are presented above graphs, and *post hoc* test statistical significance between age groups is denoted by bars with dissimilar letters above the corresponding bar (e.g. A is not different than **A**, **B**; but **A** is different than **B** at the *P* < 0.05 level)

BiP protein expression in the plantaris muscle was greater in 3 mo versus all other age cohorts except 12 mo (*P* ≤ 0.008; Fig. 1A), and 24 mo had lower BiP protein expression than 3 mo and 12 mo (*P* < 0.002). Cleaved ATF6 and ATF4 failed to reach ANOVA significance (*P* ≥ 0.062, Fig. 1B-C). Phospho/pan eIF2α was greater in 24 mo versus 3 mo and 18 mo rats (P *≤* 0.006, Fig. 1D). CHOP protein was greater in 18 mo and 24 mo versus all other age groups (*P* < 0.001), and 12 mo was greater than 3 mo and 6 mo (*P* ≤ 0.022, Fig. 1E). Phospho/pan JNK was greater in 18 and 24 mo versus all other age groups (*P* ≤ 0.002, Fig. 1F), and 12 mo was greater than 3 mo (*P* = 0.009). Finally, CHOP protein expression demonstrated a significant, moderate negative correlation with plantaris weight normalized to body weight (*P* < 0.001; Spearman’s *R*=-0.702, Fig. 1G).

### ERS markers with aging in the soleus muscle

**Fig. 2:**
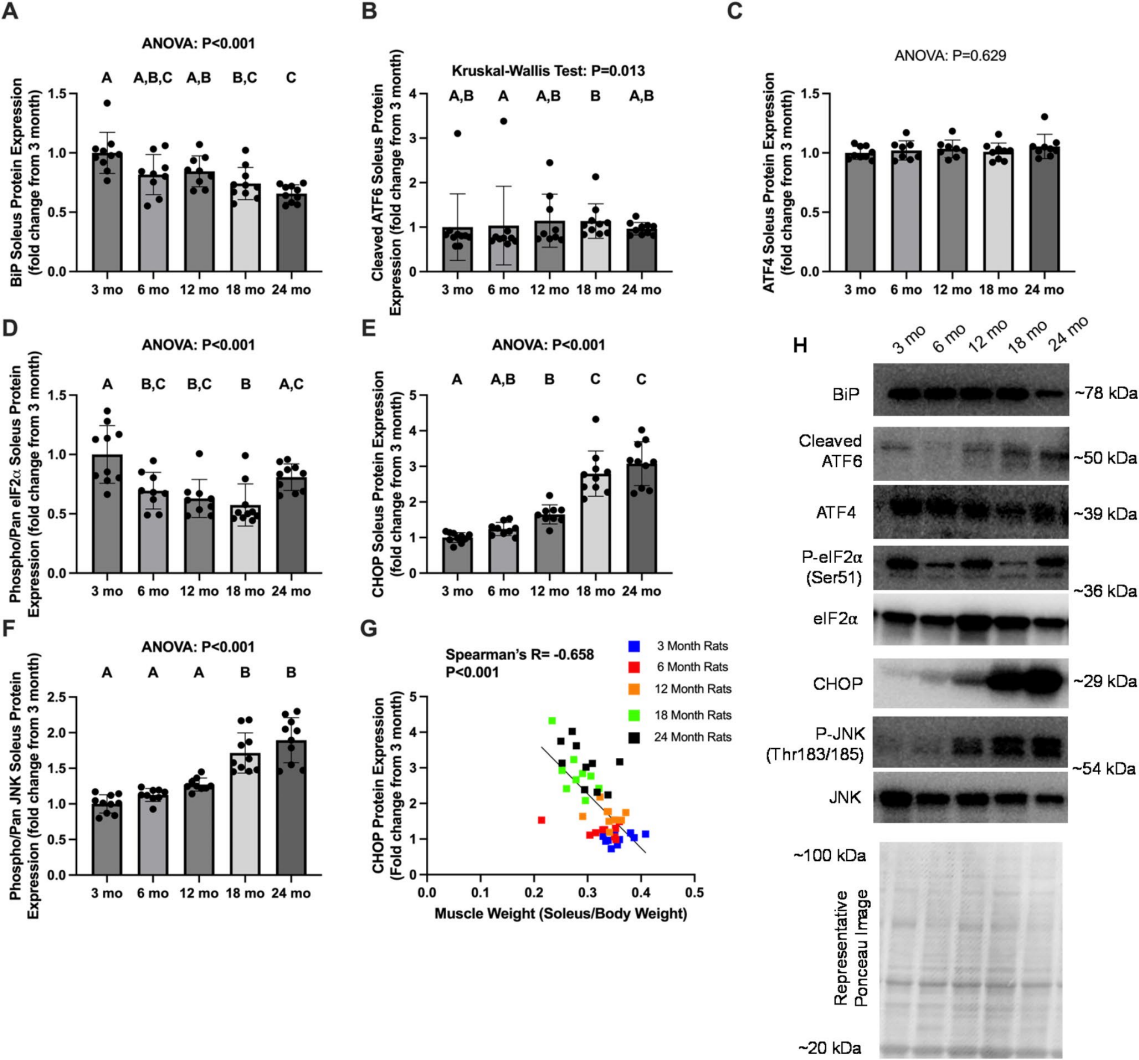
Endoplasmic Reticulum Stress related protein expression in the soleus of Fischer 344 rats with aging. Legend: Data are presented as individual data points with standard deviation as error bars (panels **A**-**F**), or individual data points with representative line of best fit (panel **G**). Results are from the soleus of Fischer 344 rats sacrificed at 3 mo (*n* = 10), 6 mo (*n* = 9), 12 mo (*n* = 9), 18 mo (*n* = 10), and 24 mo (*n* = 10) for protein expression of BiP (panel **A**), cleaved ATF6 (panel **B**), ATF4 (panel **C**), phosphorylated/pan eIF2α (panel D), CHOP (panel E), phosphorylated/pan JNK (panel **F**), and the association between CHOP and normalized muscle weight [(soleus/body weight)*100] (panel **G**). Representative western blot and Ponceau stain images are included (panel **H**). Western blot band densities are normalized to Ponceau densities. ANOVA P values are presented above graphs, and *post hoc* test statistical significance between age groups is denoted by bars with dissimilar letters above the corresponding bar (e.g. A is not different than **A**, **B**; but **A** is different than B at the *P* < 0.05 level)

BiP expression in soleus was greater in 3 mo versus 18 mo and 24 mo (*P* ≤ 0.008, Fig. 2A), and 12 mo was greater than 24 mo (*P* < 0.002). Cleaved ATF6 was greater in 18 mo and 6 mo (*P* = 0.023, Fig. 2B). ATF4 failed to reach ANOVA significance (*P* ≥ 0.073, Fig. 2C). Phospho/pan eIF2α was greater in 3 mo versus all other age groups (*P* ≤ 0.004) except for 24 mo (*P* = 0.126, Fig. 2D). CHOP protein was greater in 18 mo and 24 mo versus other age groups (*P* < 0.001, Fig. 2E), and 12 mo showed greater protein expression than 3 mo (*P* = 0.017). Phospho/pan JNK was greater in 18 and 24 mo versus other age groups (*P* < 0.001, Fig. 2F). Finally, CHOP protein expression demonstrated a significant, moderate negative correlation with normalized soleus weight normalized to body weight (*P* < 0.001; Spearman’s *R*=-0.658, Fig. 2G).

### ERS markers in gastrocnemius muscle with rat hindlimb immobilization

**Fig. 3:**
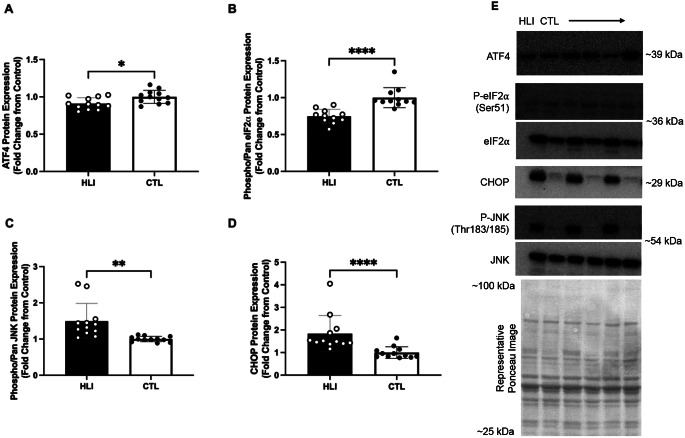
Endoplasmic reticulum stress related protein expression in the gastrocnemius of Wistar rats undergoing hindlimb immobilization. Legend: Data are presented as individual data points with standard deviation as error bars. Results are from the gastrocnemius of Wistar rats that had undergone 10 days of hindlimb immobilization (*n* = 12) or a control condition (*n* = 12) for protein expression of ATF4 (panel **A**), phosphorylated/pan eIF2α (panel **B**), phosphorylated/pan JNK (panel **C**), and CHOP (panel **D**). Representative western blot and Ponceau stain images are included (panel **E**). Western blot band densities are normalized to ponceau densities. Statistical significance is denoted by *P < 0.05, **P < 0.01, ***P < 0.001, ****P < 0.0001

Based on findings from the aging rats presented above, we opted to examine proteins that showed a consistent pattern with age, or proteins that are critical to the UPR pathway as a whole. ATF4 protein expression was higher in the gastrocnemius muscle of CTL than HLI rats (*P* = 0.014, Fig. 3A). Similarly, phospho/pan eIF2α phosphorylated protein ratio was higher in CTL than HLI rats (*P* ≤ 0.001, Fig. 3B). Phospho/pan JNK phosphorylated protein ratio was higher in HLI than CTL rats (*P* ≤ 0.002, Fig. 3C). Similarly, CHOP protein expression was higher in HLI than CTL rats (*P* ≤ 0.001, Fig. 3D).

### CHOP associations with hindlimb immobilization

**Fig. 4:**
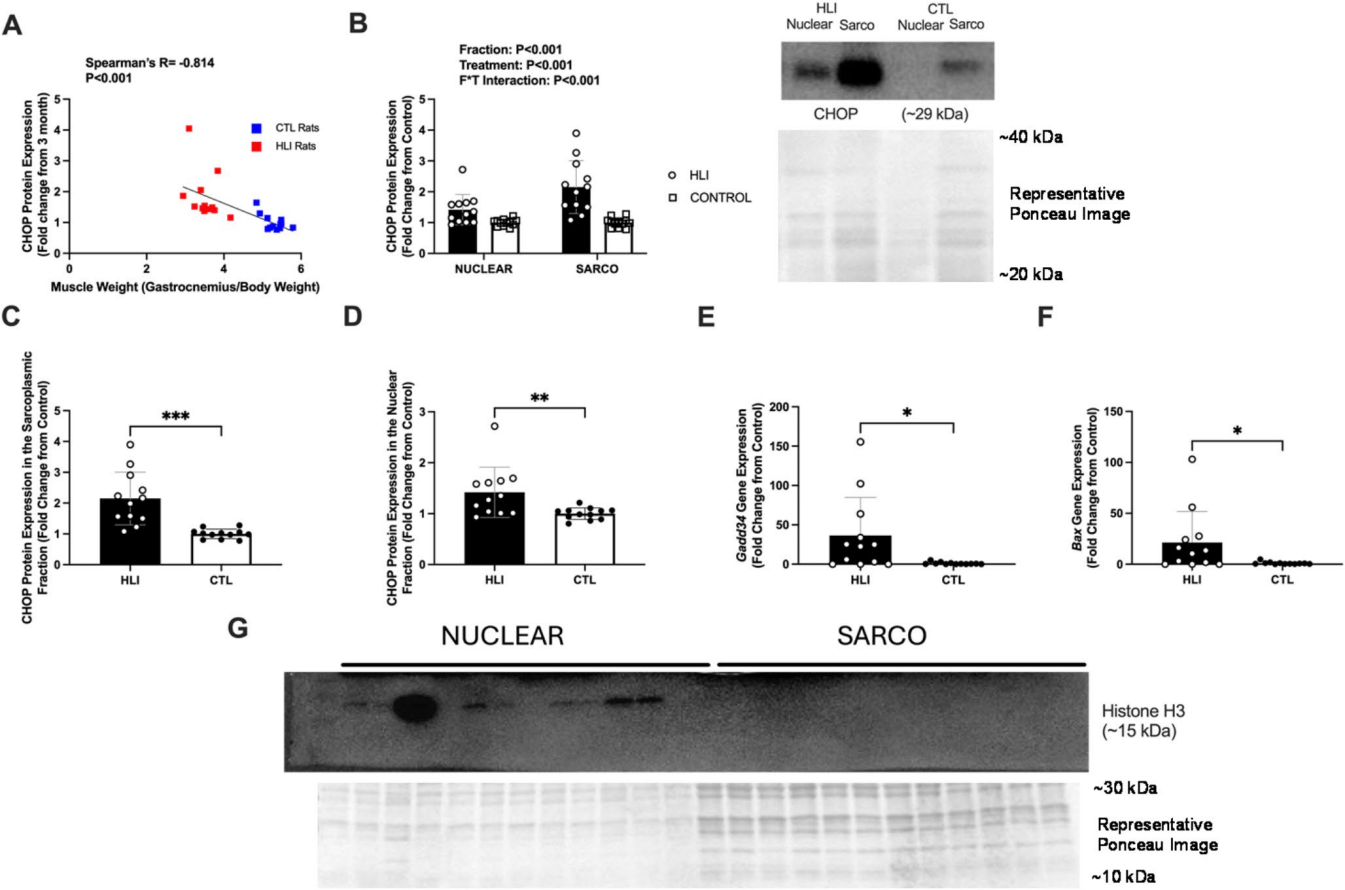
CHOP related markers from gastrocnemius muscle from Wistar rats undergoing hindlimb immobilization. Legend: Data are presented as individual points with an overlaid line of best fit (panel **A**), or individual data points with standard deviations as error bars (panels B-F). Results are from the gastrocnemius of Wistar rats that had undergone 10 days of hindlimb immobilization (*n* = 12) or a control condition (*n* = 12). Results presented are the association of CHOP with normalized gastrocnemius weight [(gastrocnemius weight/body weight)*100] (panel **A**), CHOP protein expression in the nuclear and sarcoplasmic protein fractions with representative western blot and ponceau stain image (panel **B**), CHOP expression in the sarcoplasmic fraction only (panel **C**), CHOP protein expression in the nuclear fraction only (panel **D**), *Gadd34* gene expression (panel **E**), and *Bax* gene expression (panel **F**). A validation western blot of the nuclear protein histone H3 along with a representative Ponceau stain are included to show that nuclear fractionation was successful (panel **G**). Western blot band densities are normalized to Ponceau densities

Given the robust increase in skeletal muscle CHOP protein levels with disuse (Fig. 3D) and aging (Figs. 1F and 2F), further interrogations were performed into CHOP protein and disuse atrophy. CHOP protein expression demonstrated a significant, strong negative correlation with gastrocnemius weight normalized to body weight (*P* < 0.001; Spearman’s *R*=-0.814, Fig. 4A). Upon separation of nuclear and sarcoplasmic protein fractions in HLI and CTL rats, it was found that CHOP protein expression demonstrated a main effect of protein fraction (*P* < 0.001), a main effect of treatment group (*P* < 0.001), and a protein fraction by treatment group interaction (*P* < 0.001, Fig. 4B). Subsequent unpaired t-tests were then performed to compare between treatment groups, but within protein fraction. In both the sarcoplasmic and nuclear fraction, CHOP protein expression was upregulated in HLI as compared to CTL rats (P≤, Fig. 4C-D). After finding that CHOP protein expression was higher in the nuclear fraction of the HLI rats as compared to CTL rats, targeted qPCR was performed to assess CHOP’s potential role as a transcriptional regulator. The hallmark downstream target gene of CHOP, *Gadd34* was upregulated ~ 35-fold in HLI versus CTL rats. Similarly, *Bax*, a pro-apoptotic gene, was upregulated ~ 20-fold in HLI versus CTL rats (*P* < 0.001, Fig. 4E-F). A validation western blot of the nuclear protein histone H3 is included to confirm that nuclear fractionation was successful (Fig. 4G).

## Discussion

The contributions of ERS and the UPR have been overlooked regarding the consequences of aging and disuse in skeletal muscle. The current findings demonstrate a robust upregulation of ERS and the UPR (as evidenced by upregulation of the UPR marker CHOP) at older ages in both the plantaris and soleus muscles of rats. Furthermore, these findings were recapitulated using a disuse-induced skeletal muscle atrophy model of hindlimb immobilization (HLI) in the gastrocnemius muscle of 4-month-old rats. While UPR activation was shown via CHOP upregulation, other canonical targets within the UPR pathway did not show as clear a pattern of regulation. However, it is notable that the chaperone protein BiP showed general downregulation with aging. This could serve to diminish protein folding capacity at the ER lumen and lead to enhanced UPR activation due to dissociation from the primary stress sensors PERK, IRE1α, and ATF6 [30]. This remains speculative, however, as the remaining portions of these pathways that were measured did not respond in kind. It is of note that the phosphorylated/pan protein ratio of the protein JNK responded in a similar manner to CHOP, further implicating proapoptotic cell activity in response to aging and disuse [31]. Notably, while JNK regulation can be influenced by the UPR, JNK resides in the family of mitogen activated protein kinases (MAPK) referred to as stress-activated protein kinases (SAPK) and as such have a much broader regulatory system than the UPR alone [32, 33]. Given the broad regulation of JNK through various forms of cell stress induction, we opted to focus on the more ERS associated effector CHOP. Therefore, we posit that the novel and primary finding herein is that the UPR effector CHOP is upregulated with aging and disuse in skeletal muscle.

CHOP protein expression was higher in both the soleus and plantaris muscles of 18- and 24-month-old rats versus their younger counterparts. It is intriguing that CHOP was upregulated in 18 mo rats given that others have observed an upregulation later in life [34]. To this point, the Bodine laboratory has previously demonstrated that CHOP is upregulated following disuse and subsequent reloading in aged rats [35], and in the basal state of 24 month old wild type mice [36]. In both instances, the upregulation in CHOP was linked to depressed proteasome function and an accumulation of ubiquitin tagged proteins. CHOP was additionally upregulated in the mixed gastrocnemius muscle following 10-days of HLI in rats. This finding is notable, given the marked disagreement with Hunter et al. [37] who found no upregulation of CHOP with 1- or 7-days of hindlimb unloading (HU) in Wistar rats. This investigation did, however, show expression differences between the predominantly slow-fiber soleus and the predominantly fast-fiber extensor digitorum longus (EDL), with the EDL demonstrating greater values with or without HU. Conversely, we report no difference in relative expression patterns between the slow-twitch SOL or the fast-twitch PLT with aging, and an upregulation in expression with HLI in the mixed gastrocnemius. Additionally, we found that CHOP expression negatively correlates to normalized muscle weight in all of the aforementioned muscle groups. This is a potential avenue for further investigation, as unraveling proteolytic mechanisms between fiber types is an emerging area of current research [38, 39]. Nonetheless, UPR activation via an upregulation in CHOP has been observed amongst late-age and disuse-atrophy models [26, 40]. While the exact cause for CHOP upregulation in these models is unclear, it is likely that CHOP induction is a function of an accumulation of protein aggregates due to impaired proteostasis in disuse and/or aging. To this end, it has been shown that protein aggregates and impaired proteostasis are seen in both aging and disuse models by way of impaired proteolytic systems, altered protein synthesis rates, or both [41–43]. In a model taking both aging and disuse in to account, Fuqua et al. [44] report that collagen protein synthesis was not different between 10-month and 28-month F344BN rats after 14 days of HU and 60 days of reloading. These investigators did, however, observe higher collagen concentration and an accumulation of advanced glycation end products which are indicative of impaired protein clearance (e.g. proteolysis) and overall proteostasis. This is certainly a possible mechanism by which the protein folding machinery is stressed to the point that the UPR is activated, and the end-effector CHOP is induced.

Canonically, CHOP primarily serves as an apoptotic regulator and contributing to cellular dysfunction [15, 16]. To this end, various tissue models have observed an apoptotic response when CHOP is overexpressed and a reduction/resistance to apoptosis when CHOP or its major dimerization partner C/EBPβ is knocked down or depleted [17]. While these examples are convincing, most arise from non-skeletal muscle models and tissue types. Indeed, the mechanism and existence of apoptosis in skeletal muscle (a largely post-mitotic tissue) has been debated and the true function of apoptosis in skeletal muscle remains elusive (reviewed in [45]). Notwithstanding, the present data suggest a role for CHOP in apoptotic signaling with HLI in Wistar rats. Notably, after 10-days of HLI, CHOP localization in the nuclear fraction was higher versus CTL rats, and the downstream transcriptional target of CHOP, *Gadd34*, along with the canonical apoptotic gene *Bax*, were both ~ 25-fold greater in HLI versus CTL rats. The GADD34 protein has been shown to increase in atrophic murine models of sepsis and cancer cachexia [46, 47], as well as in an in vitro model of atrophic sepsis (LPS administration) [47]. Critically however, when ERS was globally blocked with 4-phenyl butyric acid (PBA), atrophy was exacerbated in wildtype and tumor bearing mice, suggesting that there is a beneficial role of ERS in the maintenance of skeletal muscle in certain conditions [46]. In a murine model of exercise preconditioning [48] to combat HU-induced atrophy, *Gadd34* mRNA was elevated upon 1- and 3-days of HU in the control group. Interestingly, 7 days of exercise preconditioning blunted this increase at 1-day of HU but numerically exacerbated this increase at 3 days. Further, 7 days of exercise preconditioning abrogated the atrophy that was seen with 3 days of HU. Although the model of atrophy differs drastically, the peculiar effects of ERS, particularly by way of GADD34 accumulation at the protein and/or mRNA level, on skeletal muscle remodeling remain at the forefront. While the current study does not delineate between nuclear subpopulations (myonuclei versus resident stromal cells), our data imply that CHOP is operative as a transcriptional regulator with HLI. However, given the results yielded from the aforementioned studies, whether CHOP and its downstream effectors are mechanistic drivers, responsively induced, or have a compensatory role in such models of disuse atrophy remains to be determined.

The elevation in CHOP protein expression observed with aging muscle (plantaris and soleus) and HLI muscle (gastrocnemius), and the negative correlations in CHOP protein expression with muscle masses, also warrant discussion. A similar relationship has also been reported whereby the mRNA expression of several key factors in the ERS pathway were significantly correlated with skeletal muscle mass following disuse atrophy in mice [26]. These investigators also demonstrated that a global blockade of ERS (via Tauroursodeoxycholic Acid, TUDCA) mitigates skeletal muscle atrophy following 14 days of HU. While these findings implicate a role for ERS and the UPR in unloading-induced skeletal muscle atrophy, the precise signaling mechanism by which atrophy is alleviated remains unclear. Given the current data showing an upregulation of CHOP expression at later ages and in disuse atrophy, an increased nuclear localization of CHOP following HLI, and the upregulation of known downstream genes affected by CHOP with HLI, we posit that CHOP could play a key role in aging- and disuse-induced skeletal muscle atrophy. However, further mechanistic investigations into the direct role of CHOP are likely needed to fully elucidate this hypothesis.

### Limitations

We are limited in this study by the sole reliance on rodent findings and therefore cannot be certain that the findings presented herein are recapitulated in humans, but the findings could be used for the development of hypotheses to test in humans. Therefore, future research involving human disuse and/or aging models aiming to measure ERS related proteins and responses are warranted. Moreover, given that we lack data from very old rodents (e.g. 28 + months old), we cannot firmly conclude that observed responses are further affected with advanced aging in rodents. In addition to this, we were limited to the use of male rats to examine aging and female rats to examine hindlimb unloading. While sex differences likely exist and play a role in the signaling responses observed herein, it has been reported that both male and female rats experience significant atrophy in response to aging and hindlimb disuse [49, 50]. Finally, we did not perform experiments related to the experimental knockdown of CHOP in vitro or in vivo. Thus, further mechanistic investigation will be needed to determine whether CHOP is a key mechanistic driver of skeletal muscle atrophy.

## Conclusions

ERS and the UPR are upregulated with aging in rats and this response was recapitulated at a young age after 10 days of HLI in 4-month-old adult rats as evidenced by an upregulation in the end-effector protein, CHOP. In addition to global upregulation, nuclear CHOP localization and the production of mRNAs induced by CHOP’s transcriptional regulation are heightened during hindlimb immobilization induced skeletal muscle atrophy. Finally, CHOP protein expression is negatively correlated with muscle masses in aging and disuse models of atrophy. Taking these findings in total, it appears that the regulation of CHOP is sensitive to multiple atrophy inducing models. Within these systems CHOP could be atrophy responsive or atrophy inducing, however further interrogation using viral or genetic manipulation of CHOP is needed to determine this. In sum, these findings warrant additional investigation to determine if CHOP is a key mechanistic player in these two different types of skeletal muscle atrophy.

## Data Availability

The data that support the findings of this study are available from the corresponding author (mdr0024@auburn.edu) upon reasonable request.
